# Impact of Nutrition on Somatotroph Axis: A Potential Role in Acromegaly and Its Cardiovascular Risk?

**DOI:** 10.1210/jendso/bvaf195

**Published:** 2025-11-28

**Authors:** Angelo Milioto, Federico Gatto, Ingrid Larsson, Annamaria Colao, Diego Ferone, Gudmundur Johannsson, Daniela Esposito

**Affiliations:** Department of Internal Medicine and Clinical Nutrition, Sahlgrenska Academy, University of Gothenburg, Gothenburg 41346, Sweden; Department of Endocrinology, Sahlgrenska University Hospital, Gothenburg 41346, Sweden; Endocrinology Unit, Department of Internal Medicine and Medical Specialties, School of Medical and Pharmaceutical Sciences, University of Genova, Genoa 16132, Italy; Endocrinology Unit, IRCCS Ospedale Policlinico San Martino, Genoa 16132, Italy; Department of Internal Medicine and Clinical Nutrition, Sahlgrenska Academy, University of Gothenburg, Gothenburg 41346, Sweden; Department of Medicine, Sahlgrenska University Hospital, Gothenburg 41346, Sweden; Unità di Endocrinologia, Diabetologia e Andrologia, Dipartimento di Medicina Clinica e Chirurgia, Università Degli Studi di Napoli Federico II, Naples 80131, Italy; Unità di Endocrinologia, Diabetologia e Andrologia, Centro Italiano per la Cura e il Benessere del Paziente con Obesità (C.I.B.O.), Azienda Ospedaliera Universitaria Federico II, Naples 80131, Italy; Cattedra Unesco “Educazione Alla Salute E Allo Sviluppo Sostenibile,” University Federico II, Naples 80131, Italy; Endocrinology Unit, Department of Internal Medicine and Medical Specialties, School of Medical and Pharmaceutical Sciences, University of Genova, Genoa 16132, Italy; Endocrinology Unit, IRCCS Ospedale Policlinico San Martino, Genoa 16132, Italy; Department of Internal Medicine and Clinical Nutrition, Sahlgrenska Academy, University of Gothenburg, Gothenburg 41346, Sweden; Department of Endocrinology, Sahlgrenska University Hospital, Gothenburg 41346, Sweden; Department of Internal Medicine and Clinical Nutrition, Sahlgrenska Academy, University of Gothenburg, Gothenburg 41346, Sweden; Department of Endocrinology, Sahlgrenska University Hospital, Gothenburg 41346, Sweden

**Keywords:** nutrition, acromegaly, metabolism, morbidity, biochemical control, cardiovascular risk

## Abstract

Diet composition and energy intake directly modulate the growth hormone (GH) and insulin-like growth factor 1 (IGF-1) axis, and indirectly through endogenous regulators such as insulin, ghrelin, and adipokines. Moreover, diet has a well-established role in the prevention and management of various metabolic and cardiovascular comorbidities in the general population.

Acromegaly, caused by an endogenous overproduction of GH, is an endocrine disorder associated with increased risk of metabolic and cardiovascular comorbidities and excess mortality. The treatment of acromegaly aims to normalize GH and IGF-1 levels, manage complications, and reduce mortality.

There is a considerable gap in research regarding the specific influence of diet on biochemical control and complications in acromegaly; therefore, consensus guidelines for managing metabolic and cardiovascular complications in acromegaly generally recommend the same nutritional interventions as those for the general population, even though the underlying pathogenic mechanisms often differ.

This narrative review aims to provide an overview of how nutrition modulates the GH/IGF-1 axis and to summarize current evidence on the effect of various macronutrients and dietary patterns on biochemical control and comorbidity management in patients with acromegaly.

Evidence in healthy individuals suggests that diets low in animal-derived proteins, combined with moderate fat and carbohydrate intake, may lower GH/IGF-1 activity. Nevertheless, further research is needed in patients with acromegaly to determine whether dietary interventions, such as those found effective in the general population, can help achieve biochemical control and effectively manage metabolic and cardiovascular comorbidities in this specific group.

The secretion of growth hormone (GH) and its mediator insulin-like growth factor 1 (IGF-1) is controlled by the hypothalamus through growth hormone–releasing hormone (GHRH) and somatostatin release [1]. Circulating IGF-1 is bound to insulin-like growth factor binding proteins (IGFBPs), which is an additional mechanism to regulate IGF-1 availability and tissue exposure [2].

Several hormones influence GH and IGF-1 secretion [3] (Fig. 1). Oral glucose administration in healthy individuals increases insulin and glucose concentrations and reduces GH secretion [1]. Studies in rats, mice, and baboons indicate that insulin—independently of hyperglycemic conditions—suppresses GH secretion, possibly through inhibition of GH secretory vesicle release [4-6]. However, in humans, insulin-induced hypoglycemia is a robust trigger for the GH release [7]. In vitro, insulin enhances production and surface translocation of the GH receptor (GHR) in human hepatic cells, increasing liver GH sensitivity [8]. Beyond insulin, ghrelin also influences GH secretion. Ghrelin, mainly produced during fasting in the stomach [9], stimulates GH production via GH-secretagogue receptor 1a located in the anterior pituitary and hypothalamus [10, 11]. Additionally, adipose tissue influences the somatotroph axis through different adipose tissue–related hormones, known as adipokines. Studies suggest that leptin [12-14] and resistin [15, 16] increase GH secretion, whereas the effect of adiponectin on the somatotroph axis is not clear. One in vitro study found that adiponectin increases GH production in rat pituitary cells [17], whereas other in vitro studies in rats and nonhuman primates reported a reduction in GH concentration due to adiponectin administration [15, 18].

**Figure 1.**
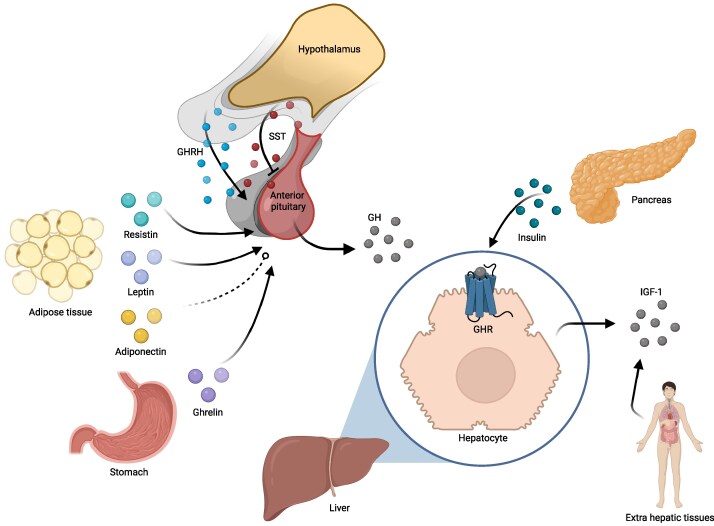
Somatotroph axis regulation by other hormones. Dotted arrow: The available evidence on the effect of adiponectin on somatotroph axis is limited and conflicting. GH, growth hormone; GHR, growth hormone receptor; GHRH, growth hormone–releasing hormone; IGF-1, insulin-like growth factor 1; SST, somatostatin.

Acromegaly is caused in more than 95% of cases by a GH-secreting pituitary adenoma [19] and is characterized by chronic exposure to supraphysiological GH and IGF-1 levels, resulting in a higher incidence of metabolic diseases—such as diabetes mellitus (DM) and dyslipidemia—as well as cardiovascular disease and other comorbidities [20]. Due to the prolonged GH excess, patients with acromegaly have a unique metabolic profile characterized by insulin resistance despite reduced adiposity and increased muscle mass [21-23]. Pituitary surgery is the first-line treatment for acromegaly but approximately 50% of patients do not achieve biochemical remission and require medical therapy with somatostatin receptor ligands (SRLs), GHR antagonists, dopamine agonists, or a combination of those [24, 25]. Despite advances in surgery and medical therapies, patients with acromegaly still have an increased standardized mortality ratio compared to the general population, likely due to diagnostic delay, difficulties in achieving and maintaining biochemical control, as well as an increase cardiovascular risk due to persistent metabolic and cardiovascular comorbidities [26-28].

In normal physiology, insulin and GH play a pivotal role in the homeostasis of macronutrients and, conversely, diet also influences GH secretion [29]. After a meal, insulin directs macronutrients toward anabolic processes such as glycogen, lipid, and protein synthesis [3]. During fasting, GH promotes lipolysis over carbohydrate oxidation and preserves protein reserves [29].

The interplay between the somatotroph axis, its regulators, and nutrition suggest the potential benefit of nutritional interventions to comprehensively manage acromegaly, especially considering the increased cardiovascular risk in these patients. However, there is a notable lack of data on how diet affects acromegaly's biochemical control and complications. The aim of this narrative review is to summarize the nutritional regulation of the GH/IGF-1 axis and synthesize the evidence on how macronutrients and different dietary regimens affect biochemical control and comorbidity management in acromegaly.

## Effect of Nutrition on Growth Hormone/Insulin-like Growth Factor-1 Axis Under Physiological Conditions

**Figure 2.**
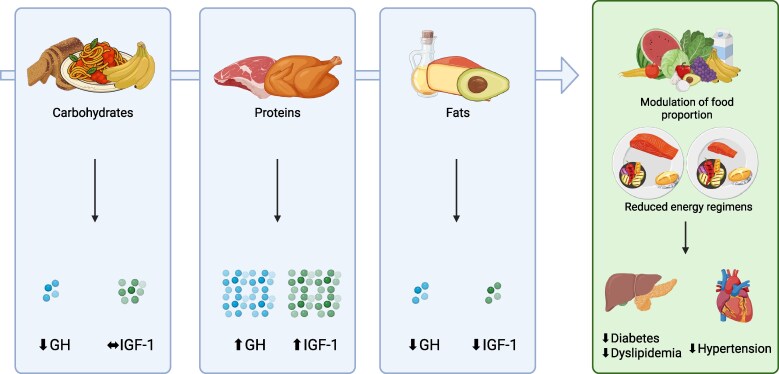
Dietary intervention and their systemic effects in general population. Macronutrients intake differentially affects growth hormone (GH) and the insulin-like growth factor 1 (IGF-1) axis: carbohydrates reduce GH without affecting IGF-1; proteins increase both GH and IGF-1; fats reduce both. Modulation of food proportion and reduced energy regimens improve different metabolic and cardiovascular comorbidities.

GH secretion is influenced both by fasting and feeding, with different effects depending on the macronutrient composition of meals consumed, which highlights the interplay between nutritional intake and the GH/IGF-1 axis [30] (Fig. 2).

### Interaction Between Macronutrients and Growth Hormone/Insulin-like Growth Factor-1

#### Carbohydrates

Glucose is a monosaccharide that represents a key energy source for most cells and influences GH concentration. Indeed, hyperglycemia suppresses GH secretion, whereas hypoglycemia stimulates it [7, 31]. Although the exact mechanism remains unclear, glucose appears to promote hypothalamic somatostatin release, which inhibits GH production [32, 33].

Total carbohydrate intake also affects GH secretion during dietary interventions. In a study conducted on 8 men and 7 women, the mean sum of 24-hour GH concentrations (assessed using hourly sampling) decreased following a 10-day high-carbohydrate diet, isocaloric or hypercaloric, but only in men [34]. Carbohydrate intake also stimulates insulin secretion; in vitro evidence indicates that insulin upregulates both the production and surface translocation of hepatic GHR [8]. Accordingly, in women with obesity treated with recombinant human GH, those following an energy-restricted, high-carbohydrate diet (80% of energy [E%]) had a greater IGF-1 than those on an energy-restricted, high-fat diet (72 E%), with equivalent E% of protein in both groups [35]. Ghrelin levels decrease after carbohydrate ingestion [36-38], whereas leptin secretion is increased [39, 40]. Similarly, in healthy individuals, an inverse correlation has been observed between adiponectin and dietary carbohydrates [41-44]. Lastly, one study conducted on 36 individuals has shown that resistin levels are suppressed during an oral glucose tolerance test [45].

#### Proteins and amino acids

Proteins are a class of macronutrients involved in various physiological functions, and amino acids are the primary constituents of proteins [46]. The effect of proteins and amino acids on GH secretion has been extensively studied.

Oral amino acid intake raises circulating GH levels 2- to 8-fold above baseline, depending on the specific amino acid, age, and physical activity of the individual [47, 48]. For example, arginine is widely used intravenously with GHRH during a stimulation test of the somatotroph axis in patients with suspected GH deficiency [49]. Oral administration of arginine to 6 healthy men increased integrated GH concentrations by 2.8 times compared to placebo over 5 hours [50]. High-protein diets also stimulate GH release [51]. A large cross-sectional study demonstrated that a vegan diet is associated with lower IGF-1 and higher IGFBP-1 and IGFBP-2 levels compared to a vegetarian diet and a meat-based diet, suggesting a stronger effect of animal-derived proteins compared with plant-derived proteins on the GH/IGF-1 axis [52]. A high-protein diet acutely increases insulin [53], whereas no effect on leptin has been demonstrated [54]. The effect of dietary proteins on adiponectin and resistin remains undefined, although amino acid supplementation increases adiponectin transcription in human visceral adipocytes [55]. Finally, high-protein meals reduce ghrelin levels [37, 38, 56-58]. In summary, evidence suggests that high protein intake is associated with greater somatotroph axis activity, increased insulin, and decreased ghrelin, while the effects on adiponectin and resistin remain uncertain.

#### Fats and fatty acids

Fats are a heterogeneous group of compounds that serve as a major energy source. Fatty acids are structural components of cell membranes and precursors for steroid hormones [59]. During fasting, GH enhances lipolysis raising the circulating free fatty acids (FFAs) [29]. Conversely, FFAs reduce GH secretion [60], likely through inhibition of somatotroph cells [60] and an increase of somatostatin release in the hypothalamus [61]. A meta-analysis of randomized clinical trials (RCTs) demonstrated that replacing carbohydrates with fats in an isocaloric diet reduces postprandial insulin levels, regardless of FFA concentration [62]. Dietary fat reduces ghrelin [37, 38, 63] but does not appear to affect adiponectin [64, 65] and resistin [66]. Finally, a reduction of leptin following dietary fat has been observed [67, 68]. Ultimately, dietary fats decrease GH/IGF-1 axis activity, likely by raising FFAs and concurrently reducing insulin, ghrelin, and leptin levels.

### Fasting and Reduced Energy Intake Regimens

In recent decades, various dietary protocols focusing on the modulation of energy intake and timing have been studied for disease prevention, with some benefits linked to their effects on the GH/IGF-1 axis [69].

GH mobilizes endogenous energy sources, particularly during fasting [29]. Water-only fasting for 36 to 48 hours increase GH secretion in healthy individuals, while IGF-1 remains unchanged or decreases [70-72]. Of note, an increase in IGFBP-1, and therefore a reduction in IGF-1 bioavailability, has been demonstrated in 2 studies [70, 72]. On the other hand, observational studies of prolonged starvation such as in patients with anorexia nervosa report elevated GH with reduced IGF-1 concentrations [73].

Energy restriction (ER) is defined as a reduction in energy intake compared with the usual diet that meets essential micronutrient and macronutrient needs, preventing malnutrition [74]. In a 6-day trial of ER involving 8 participants, IGF-1 decreased and IGFBP-1 increased; 3 days after refeeding, IGF-1 rose but remained below baseline, while IGFBP-1 normalized [75]. A 12-month long study on ER (16% for 3 months, then 20% for 9 months) and high protein intake in 18 normal-weight individuals showed stable IGF-1 and IGFBP-3 levels [76]. In a later phase, a subgroup of 6 participants underwent ER with reduced protein intake for 3 weeks, resulting in a decrease in IGF-1 but stable IGFBP-3 levels [76]. Therefore, ER alone may not be able to induce long-term lowering in IGF-1 unless accompanied by a reduction in protein intake.

The fasting-mimicking diet (FMD) is a plant-based regimen designed to reproduce the metabolic effects of water-only fasting while mitigating undernutrition, typically providing 300 to 1100 kcal per day [77]. In an RCT of 100 healthy individuals, participants followed either their usual diet or a 5-day FMD each month for 3 months [78]. The FMD group demonstrated lower IGF-1 compared to the control group, with an initial increase in IGFBP-1 that normalized after 3 cycles. Participants with higher baseline IGF-1 showed a greater and more sustained decrease, with low IGF-1 persisting 3 months after the trial ended [78].

Intermittent fasting (IF) includes various dietary strategies alternating fasting periods—typically at least 12 hours of water-only fasting or severe ER—with periods of ad libitum intake [69]. In a non-RCT, 22 healthy participants underwent IF for 8 weeks (1 fasting day per week, with up to 300 kcal/day from liquids); no significant changes in IGF-1 were observed in the fasting group, either compared to baseline or to a control group maintaining their usual diet [79]. Conversely, a study on resistance-trained men undergoing 8 weeks of daily 16-hour fasting and 8-hour feeding showed reduced IGF-1, insulin, and fat mass from baseline, compared to a control group [80]. In conclusion, there are discrepant findings on the effect of IF on IGF-1; therefore, further studies with homogeneous protocols, populations, and dietary compositions are needed.

## Dietary Modulation and Energy Restriction for Biochemical Control in Acromegaly

Investigating the relationship between the somatotroph axis and nutrition in acromegaly could provide insights on how diet can potentially complement conventional therapies to achieve biochemical remission. Next, 3 studies examining the effects of the modulation of food proportion and total ER on GH/IGF-1 axis are reviewed, with a summary presented in Table 1.

**Table 1.** Evidence on the effect of different dietary and energy restriction interventions on biochemical control in acromegaly

Study	Study design	Study population	Intervention	Result
**Ho et al, 1992** [81]	Clinical trial	7 patients with active acromegaly, 6 control individuals	Water only fasting for 6 days	↓ IGF-1 in patients with acromegaly (significant after 5 d)
				↓ IGF-1 in control individuals (significant after 4 d)
				↔ GH in patients with acromegaly
				↔ GH in control individuals
**Coopmans et al, 2020** [82]	Clinical trial	11 patients with active acromegaly treated with first-generation somatostatin ligand	Eucaloric very-low carbohydrate*^a^* diet for 2 wk	↓ IGF-1
				↔ GH
**Grottoli et al, 2008** [83]	Clinical trial	8 patients with active acromegaly, 7 control individuals	Water only fasting for 36 h	↔ IGF-1 in patients with acromegaly (trends toward reduction)
				↓ IGF-1 in control individuals
				↔ GH in patients with acromegaly
				↔ GH in control individuals

Abbreviations: GH, growth hormone; IGF-1, insulin-like growth factor 1.

^a^A total of 35 g of carbohydrates per day.

An experimental trial included 7 patients with active acromegaly (6 treatment naive, 1 post surgery) and 6 healthy volunteers. All participants underwent 6 days of water-only fasting in an inpatient setting, with IGF-1 and GH monitored throughout [81]. Serum IGF-1 decreased after 5 days in the acromegaly patients and after 4 days in controls. By day 6, IGF-1 dropped to 65% of baseline in the acromegaly patients and 50% in controls. On the other hand, GH levels did not change in the acromegaly patients but increased in the healthy individuals [81].

Coopmans and colleagues [82] conducted a clinical trial in 11 patients with active acromegaly treated with a first-generation SRL (1g-SRL) who followed a eucaloric very-low-carbohydrate ketogenic diet (35 g/day) for 2 weeks. Serum IGF-1 decreased from 1.1 × upper limit of normal (ULN) to 0.83 × ULN, falling into the normal range in all but 1 patient while no significant changes in GH were observed. In a subgroup of 6 patients, a eucaloric low-carbohydrate ketogenic diet (80 g/day) for 3 months led to sustained IGF-1 reduction, with 5 patients achieving normal IGF-1 after 3 months. Notably, 1g-SRL doses were reduced in 3 cases [82].

In another study, the effects of 36 hours of fasting on metabolism were studied in 8 women with active acromegaly who had received no previous treatment, and 7 healthy women [83]. After fasting, GH and IGF-1 levels in acromegaly patients were unchanged, whereas healthy individuals showed increased GH and decreased IGF-1. Insulin levels decreased in both groups but remained higher in the acromegalic group than controls [83].

Evidence indicates that both total ER and eucaloric ketogenic diets reduce IGF-1, likely due to lower portal insulin and decreased hepatic GH sensitivity [81-83]. Notably, a longer fasting period might be necessary to observe this effect in patients with acromegaly compared to healthy individuals, potentially to overcome the insulin resistance in the liver observed in this population. However, implementing prolonged total ER in clinical practice is challenging due to safety concerns like malnutrition and poor long-term adherence [73, 84]. Moreover, the possibility that fasting could enhance GH secretion from remaining tumor tissue remains unclear. Considering these limitations, alternative dietary strategies with a more favorable safety and adherence profile—such as eucaloric ketogenic diet and short-term ER—should be explored as potential adjuncts to conventional therapies in acromegaly.

## Nutritional Intervention in the General Population to Manage Conditions Overlapping With Acromegaly Comorbidities

The role of diet is well established in the prevention and management of highly prevalent diseases in the general population, such as dyslipidemia, DM, and arterial hypertension (AH) (see Fig. 2). A recent nationwide study conducted in Sweden demonstrated that cardiovascular diseases represent the leading cause of mortality among patients with acromegaly [27]. Dyslipidemia, DM, and AH are 3 of the major metabolic complications of acromegaly and represent modifiable risk factors for cardiovascular events [85-87].

### Dyslipidemia

Dyslipidemia is frequent in acromegaly, affecting 13% to 51% of patients [88-92]. Under physiological conditions, GH enhances lipolysis during fasting, thereby increasing FFAs, favoring lipids as a preferential energy substrate, and protecting the protein reserves [29]. Under conditions of GH excess, increased lipolysis and reduced lipoprotein lipase activity contribute to dyslipidemia [85, 90, 93-95]. Specifically, patients with acromegaly have higher triglycerides and lower high-density lipoprotein, while total cholesterol and low-density lipoprotein are similar to or increased compared with those of healthy individuals [93, 94]. Guidelines for dyslipidemia recommend increasing the intake of vegetables, legumes and fruits, unsaturated fats, whole-grain and fiber-rich cereals, and reducing saturated fats, red meat, processed meat, added sugar, sodium, and alcohol [96, 97]. These dietary changes improve lipid profile and reduce cardiovascular risk in the general population.

### Diabetes Mellitus

The prevalence of DM is increased in patients with acromegaly [98], attested in 30% [99-101]. Furthermore, DM often persists even after biochemical control, with rates varying by treatment type [85, 100, 102]. Notably, patients with acromegaly and associated DM have increased overall mortality, as well as increased cardiovascular mortality and morbidity compared to patients with acromegaly without DM [103]. GH promotes insulin resistance both directly—by downregulating insulin signaling—and indirectly by increasing FFAs, which contend with glucose for uptake due to substrate competition at the Krebs cycle [104, 105]. Unlike the general population, in which insulin resistance is linked to increased adiposity, GH-induced insulin resistance in acromegaly occurs with reduced body fat due to GH lipolytic effects [106].

Different dietary regimens have been demonstrated to effectively contribute to the management of DM, especially type 2 DM [107, 108]. A meta-analysis of RCTs including patients with type 2 DM demonstrated that a very-low-carbohydrate (<10% E%) ketogenic diet and low-carbohydrate diets (<26 E%) induce greater short-term reductions in glycated hemoglobin A_1c_ (HbA_1c_) than high-carbohydrate diets (>45 E%), although the effect is not maintained long term [109]. Ketogenic diet and low-carbohydrate diets also lead to greater weight loss, suggesting HbA_1c_ improvements may be partly due to weight reduction [109]. Studies on ER in type 2 DM, though limited by small sample sizes and duration, show increased weight loss but no HbA_1c_ improvement when compared to nonfasting approaches [110, 111]. Interestingly, an RCT comparing IF and ER found both significantly reduced HbA_1c_ and body weight without either showing superiority over the other in either outcome [112]. Of note, adopting a new diet often leads to weight loss, which improves glucose metabolism [113]. Other factors like physical activity also improve glycemic control in DM, regardless of diet [114], but these are beyond the scope of this review.

### Arterial Hypertension

AH is a frequent complication in acromegaly, affecting 35% of patients [115]. Chronic GH/IGF-1 excess increases myocardial contractility, cardiac output, sodium and fluid retention, and heart rate, leading to AH [116-119]. AH often persists even after biochemical control of acromegaly, highlighting the need for long-term cardiovascular risk management in these patients [101].

Dietary interventions play a pivotal role in the management of AH [120]. Excessive sodium consumption is linked to elevated blood pressure; thus, reducing dietary sodium intake is a crucial approach in the management of AH [121]. A meta-analysis of RCTs showed that modest sodium reduction lowers systolic blood pressure by 5 mm Hg in patients with AH [122]. The Dietary Approaches to Stop Hypertension (DASH) diet—rich in fruits, vegetables, and low-fat dairy products, and low in saturated fats, cholesterol, sodium, and alcohol—has been demonstrated to reduce systolic blood pressure by 11 mm Hg in patients with AH [123]. A recently published RCT found that combining DASH with IF led to a greater reduction in systolic blood pressure after 3 weeks and greater weight loss after 5 weeks than DASH alone [124]. However, since blood pressure improvement occurred before weight loss, this effect may be independent of weight reduction [124].

While dietary interventions effectively manage AH in the general population, their effect on patients with acromegaly and AH remains unexplored. Since GH promotes sodium retention, nutritional interventions based on sodium intake reduction may represent an effective approach for patients with acromegaly and AH. A recent study conducted on patients with primary hyperaldosteronism—also characterized by sodium retention—showed that sodium restriction results in a reduction in blood pressure [125].

## Future Perspectives

Four clinical trials are currently investigating the effect of different nutritional approaches in acromegaly.

Preliminary results from a clinical trial (NCT07100587) that enrolled 25 patients with acromegaly—all receiving ongoing treatment with 1g-SRL, pegvisomant, or both—were presented at the last European Congress of Endocrinology [126]. Patients sequentially underwent 2 dietary interventions, each lasting 3 weeks: first a ketogenic diet (70 E% fat, 25 E% protein, 5 E% carbohydrate), followed by a Mediterranean diet (40 E% carbohydrate, 30 E% protein, 30 E% fat). After the ketogenic diet, reductions from baseline were observed in IGF-1, glycemia, fat mass, lean mass, body weight, body mass index, as well as in waist and hip circumference. In contrast, during the Mediterranean diet, glucose, insulin, and insulin resistance—derived from the homeostasis model assessment index—increased compared with the ketogenic phase. These findings suggest that a ketogenic diet may decrease IGF-1 levels, potentially through insulin reduction, and improve several metabolic parameters in patients with acromegaly.

An ongoing randomized 3-arm trial (NCT05401084) is enrolling patients with acromegaly to compare an 8-week ketogenic diet, a low-gluten diet, and continuation of the usual diet, evaluating biochemical disease control and quality of life. Another ongoing randomized 2-arm trial (NCT06949891) is enrolling patients with treated acromegaly and IGF-I greater than 0.8 × ULN to compare a eucaloric ketogenic diet for 3 months followed by a less-restrictive eucaloric ketogenic diet for another 3 months vs a eucaloric Mediterranean diet. Finally, a single-arm trial protocol (NCT05298891) has been published and aims to investigate the effects of a low-protein diet in patients with acromegaly receiving 1g-SRL, but recruitment has not yet started.

The results of these short- and medium-term trials are expected in the coming years and may clarify the effects of low-carbohydrate, low-protein, and low-gluten diets in patients with acromegaly. However, additional data, particularly from long-term studies, are required before specific nutritional strategies can be recommended as adjuncts to conventional treatments, with the goal of implementing a holistic approach to improve biochemical control and reduce cardiovascular risk in patients with acromegaly.

## Conclusions

In recent decades, interest in diet as a determinant of health has grown substantially. Diet has a crucial role in the prevention and management of highly prevalent conditions in the general population, such as DM, dyslipidemia, and hypertension. However, evidence supporting either similar or specific nutritional intervention for the treatment of acromegaly-related comorbidities is lacking due to limited research on this issue. Therefore, investigating the effects of dietary interventions in patients with acromegaly may be particularly important, given that the excess mortality in these patients is strongly influenced by comorbidities such as hypertension and DM.

There is some evidence that macronutrients exert differential effects on the GH/IGF-1 axis, specifically with i) carbohydrates suppressing GH secretion without affecting IGF-1 concentrations; ii) protein intake increasing both GH and IGF-1; and iii) fat reducing both GH and IGF-1 concentrations. Exploratory studies conducted in patients with acromegaly demonstrated the potential role of total ER and a ketogenic diet in reducing IGF-1 levels; however, these approaches are associated with safety and adherence concerns. The available data on the effects of fasting and different macronutrients on the GH/IGF-1 axis may be used to develop a tailored diet for patients with acromegaly. This dietary intervention would aim to reduce somatotroph axis activity and contribute to the management of metabolic and cardiovascular complications, with the potential to reduce the need for medical treatment and, ultimately, reduce mortality in this population.

Ultimately, there are limited studies addressing the role of nutrition and diet as strategies for preventing or managing comorbidities associated with acromegaly, and for improving biochemical control of the disease. While waiting for the results of the ongoing clinical trials, dietary recommendations established for more prevalent related conditions—such as type 2 DM and primary AH—may be considered (Table 2). Additionally, diets with a reduced intake of animal-derived proteins, moderate fat, and carbohydrates may help achieve biochemical control, in conjunction with conventional treatments, in patients with acromegaly.

**Table 2.** Suggested dietary regimen for acromegaly comorbidities

Comorbidities	Suggested nutritional approach
**Dyslipidemia**	↑ Vegetables, fruit, legumes, nuts and seeds, whole-grain products, fish and shellfish, vegetable oils and fats, low-fat dairy products
	↓ High-fat dairy products, red and processed meat, sugar, sodium, alcohol
	Moderate energy intake or energy restriction if weight loss is needed
**Diabetes mellitus**	↑ Vegetables, fruit, legumes, nuts and seeds, whole-grain-products, fish and shellfish, vegetable oils and fats, low-fat dairy products
	↓ High-fat dairy products, red and processed meat, sugar, sodium, alcohol
	Moderate energy intake or energy restriction if weight loss is needed
**Arterial hypertension**	↑ Vegetables, fruit, legumes, nuts and seeds, whole-grain-products, fish and shellfish, vegetable oils and fats, low-fat dairy products
	↓ High-fat dairy products, red and processed meat, sugar, sodium, alcohol
	Moderate energy intake or energy restriction if weight loss is needed

This table is based on evidence obtained from studies conducted on individuals without acromegaly in the general population.

## Data Availability

Data sharing is not applicable to this article as no datasets were generated or analyzed during the current study.

## Abbreviations

1g-SRL: first-generation SRL
AH: arterial hypertension
DASH: Dietary Approaches to Stop Hypertension
DM: diabetes mellitus
E%: percentage of energy
ER: energy restriction
FFAs: free fatty acids
FMD: fasting-mimicking diet
GH: growth hormone
GHR: growth hormone receptor
GHRH: growth hormone–releasing hormone
HbA_1c_: glycated hemoglobin A_1c_
IF: intermittent fasting
IGF-1: insulin-like growth factor 1
IGFBPs: insulin-like growth factor binding proteins
RCTs: randomized clinical trials
SRL: somatostatin receptor ligand
ULN: upper limit of normal
